# Pathological right ventricular changes in synthesized electrocardiogram in end-stage renal disease patients and their association with mortality and cardiac hospitalization: a cohort study

**DOI:** 10.1186/s12882-022-02707-9

**Published:** 2022-02-24

**Authors:** Yunis Daralammouri, Jamal Qaddumi, Khubaib Ayoub, Doaa Abu-Hantash, Mai Arafat Al-sadi, Rofayda M. Ayaseh, Murad Azamtta, Osama Sawalmeh, Zakaria Hamdan

**Affiliations:** 1 Department of Cardiology, An-Najah National University Hospital, Nablus, Palestine; 2 Department of Medicine, Faculty of Medicine and Health Sciences, An-Najah National University, Nablus, Palestine; 3 Public Health Department, Faculty of Medicine and Health Sciences, An-Najah National University, Nablus, Palestine; 4 Department of Internal Medicine, An-Najah National University Hospital, Nablus, Palestine; 5 Nephrology Unit, An-Najah National University Hospital, Nablus, Palestine

**Keywords:** Right ventricle, Synthesized ECG, Mortality, Cardiac hospitalization, Volume load, Ultra filtration

## Abstract

**Background:**

Right ventricular (RV) function is an important prognostic predictor for end-stage renal disease (ESRD) patients. Non-invasive evaluation of RV function by simple electrocardiogram (ECG) is not yet evident. The purpose of this article was to investigate the presence and association of pathological right ventricular changes in synthesized ECG with cardiac hospitalization and mortality.

**Methods:**

A prospective cohort study of 137 ESRD patients (mean age: 56 years) were recruited from the hemodialysis unit in An-Najah National University Hospital, Nablus, Palestine. Synthesized ECG was done right before the hemodialysis (HD) session. The pathological right ventricular changes were recorded for each patient. The relationship between pathological RV changes and mortality, cardiac and non-cardiac hospitalization was assessed through a 6-months follow-up period.

**Results:**

Right ventricular Q wave was found in 2.2% of patients, while right ventricular ST elevation was found in 0.7% of patients, and right ventricular negative T wave was found in 0.7% of patients. During the 6-month period of follow-up, 36 (26.3%) patients were hospitalized, nine patients (6.6%) due to cardiac causes. A total of 8 (5.83%) patients died, out of those 4 patients (2.91%) due to cardiac causes. Using Fisher’s exact test, there was a significant association between pathological abnormalities in synthesized ECG and hospitalization among hemodialysis patients, (*p* = 0.047). Pathological changes in synthesized ECG were less prevalent in non-cardiac hospitalizations than in cardiac hospitalizations.

**Conclusions:**

The presence of pathological RV synthesized ECG changes can predict cardiac hospitalization in ESRD patients. Synthesized ECG is a good available tool that can be easily performed in ESRD patients. To determine whether Synthesized ECG can be used as a screening tool for pathological RV changes in a dialysis patients, more research with a larger number of patients and a longer follow-up period is required.

## Background

In recent years, the prevalence and incidence of end-stage renal disease (ESRD) have steadily increased worldwide [1], and Palestine is not excluded. A study was conducted in 2010 revealed that the measured prevalence of ESRD patients in West Bank was estimated to be 240.3 Per Million Population PMP [2]. Clinically, the most common complications of ESRD are cardiovascular diseases (CVD), which are considered the primary source of adverse outcomes in ESRD patients [3–5]. As a prevalent condition correlated with adverse health effects, left ventricular dysfunction (LVD) was therefore widely investigated in the field of dialysis [6, 7].

However, several studies have recently documented a significant prevalence of right ventricular dysfunction (RVD) in ESRD patients [8–10]. In addition, it has been documented that impaired right ventricle function (RVF) would result in more severely impaired left ventricle function (LVF) [11, 12]. Moreover, in the presence of RVD, a greater risk of death, arrhythmias, and cardiogenic shock has been identified [13, 14]. RVD has since been strongly correlated with significant mortality and morbidity due to CVD among the ESRD population [15]. Therefore, it is absolutely crucial for patients on regular hemodialysis (HD) to undertake a timely evaluation of RVF.

Transthoracic echocardiography is undoubtedly the most important non-invasive technique used in heart function assessment [16]. Because of its increased spatial and temporal resolution, cardiac magnetic resonance (CMR) imaging has become the preferred method for the non-invasive evaluation of RVF and size [17, 18]. However, an easy access screening test in ESRD patients is needed to predict CVD at an early stage. Electrocardiogram (ECG) is an easily obtainable, affordable, non-invasive test for early detection of CVD.

Multiple studies have examined different ECG variables and their correlation with CV events in patients on regular hemodialysis. Multiple ECG variables and irregularities have been reported, such as ischemic changes, rhythm disorders, repolarization disturbances, and left ventricular hypertrophy [19–23]. There was also a correlation between other ECG variables changes and increased adverse health outcomes in ESRD patients. These include increased heart rate, T-wave changes, PR-interval prolongation, atrial fibrillation, left ventricular hypertrophy, and long corrected QT (QTc) interval [24–32].

However, ECG variations in the right ventricle (RV) lead and their correlation with morbidity and mortality in ESRD patients have not been examined in previous studies up to the authors’ knowledge. This prospective cohort study aimed to assess the relationship between different ECG variations in RV leads and predictable cardiovascular complications among ESRD patients on regular hemodialysis.

## Methods

### Study design, settings, and population

This prospective cohort study was conducted between July 2014 and January 2015 at the dialysis unit of An-Najah National University Hospital, Nablus-Palestine. This unit is the largest center in the West Bank, with more than 350 patients receiving both hemodialysis and peritoneal dialysis therapy.

All participants were ESRD patients’ regular hemodialysis (three times weekly, 4 h per session) who had been on long-term HD therapy and used as a convenient purposive sample (180 patients). Nine patients refused to participate, and thirty patients were excluded due to heart failure, previous myocardial infarction(MI) (or had symptoms or signs of myocardial ischemia), valvular heart disease, pericardial disease, congenital heart disease, pulmonary hypertension, patients with left ventricular ejection fraction less than 40%, history of pulmonary embolism, chronic lung disease (cor-pulmonale) or arrhythmogenic right ventricular cardiomyopathy, using previous echocardiography reports, and hospital files. All causes of right ventricular dysfunction other than chronic volume overload were excluded. Patients less than 18 years were also excluded (4 patients). The final study group consisted of eligible 137 patients. All participants were provided with an oral and written consent form for participation. The study was approved by the Institutional Review Board (IRB) of An-Najah National University.

### Data collection, study procedure, and tools

Clinical and demographic characteristics were collected from the participants and their medical records. These included age, gender, number of years on dialysis, diabetic status (yes, no), hypertension status (yes, no), hospitalization, and ultrafiltration (UF) volumes were obtained directly from the patients or their hospital files.

Synthesized ECG provides further information about the RV than the standard 12 lead ECG by using added leads (V3R, V4R, V5R, and three posterior leads) representing the right side of the heart. The 12-lead ECG cannot detect pathological variations in the posterior wall and right ventricle. Different electrode sites from the regular 12-lead ECG should be used. Synthesized 18-lead ECG utilizes 12-lead ECG wave patterns to mathematically generate the output waveform of the right chest (V3R, V4R, and V5R) and the back leads (V7, V8, V9).

Included patients underwent synthesized ECG right before the onset of the hemodialysis session by well-trained staff. The presence and the total number of pathological RV changes (RV Q wave, RV ST Elevation, and RV negative T wave) were recorded for each patient. The synthesized ECG changes that indicate RV pathology were: 1) RV Q wave: Q amplitude ≥ 0.1 mV and Q duration ≥ 40 ms; 2) RV ST elevation: ST elevation ≥ 0.1 mv; and 3) RV negative T wave: T amplitude ≤ -0.2 mv. This criterion was obtained from the manual that came with the synthesized ECG device. All ECGs were read and reviewed by an experienced certified cardiologist.

### Statistical analysis

The clinical and demographic characteristics of the participants were summarized using descriptive statistics. Frequencies with percentages were used for categorical variables. Cross tabulation, Fisher’s exact test was used to examine for any statistically significant relation between characteristics of patients, mortality, and hospitalization with the occurrence of pathological changes in the synthesized ECG. Any *p*-value less than or equals 0.05 is considered statistically significant, and all analyses were conducted using the Statistical Package of Social Sciences (SPSS) computer software version 21.0 (IBM Corp).

## Results

### Baseline demographics

The baseline demographic and clinical characteristics of the patients are detailed in Table 1. One hundred thirty-seven patients were enrolled, and all of them completed the study. The mean age of the participants was 56 years (SD = 14.07) and 78 patients (56.9%) were males. Forty-four percent were between 40–59 years old. Thirty-tow percent (*n* = 44) were on dialysis for one year or less, and 67.9% (*n* = 93) were on dialysis for more than one year. About 54.7% (*n* = 75) and 69.3% (*n* = 95) were diabetics and hypertensive, respectively. The mean for UF volume was 3672 (± 999) ml, and 38.4% of the patients had their UF volume between 3000–3999 ml. Although pathological changes in synthesized ECG are higher in males 40–60 years old, dialysis duration more than 12 months, UF volume between 4000–4999, and those with a history of diabetes mellitus and hypertension, there is no significant correlation between these differences.

**Table 1:** Baseline Characteristics of the Study Population

**Variable**	**Total n (%)** ***n* = 137**	**Pathological Changes in synthesized ECG**	
		**No n (%)** ***n* = 132**	**Yes n (%)** ***n* = 5**
**Age**			
20-39y	15 (10.9)	14 (10.6)	1 (20)
40-59y	60 (43.8)	57 (43.2)	3 (60)
60-79y	60 (43.8)	59 (44.7)	1 (20)
≥ 80y	2 (1.5)	2 (1.5)	0 (0.0)
**Gender**			
Female	59 (43.1)	58 (44)	1 (20)
Male	78 (56.9)	74 (56)	4 (80)
**Duration of dialysis**			
≤ 12 months	44 (32.1)	43 (32.6)	0 (0.0)
> 12 months	93 (67.9)	89 (67.4)	5 (100)
**Diabetes**			
No	62 (45.3)	61 (46.2)	1 (20)
Yes	75 (54.7)	71 (53.8)	4 (80)
**Hypertension**			
No	42 (30.7)	40 (30.3)	2 (40)
Yes	95 (69.3)	92 (69.7)	3 (60)
**UF Volume**			
1000-1999 ml	4 (3)	4 (3)	0 (0.0)
2000-2999 ml	32 (23.3)	31(23.5)	1 (20)
3000-3999 ml	53 (38.7)	52(39.4)	1 (20)
4000-4999 ml	32 (23.3)	30(22.7)	2 (40)
5000-5999 ml	14 (10.2)	13 (9.8)	1 (20)
6000-6999 ml	2 (1.5)	2 (1.5)	0 (0.0)

*ECG* Electrocardiogram, *UF* Ultra filtration

### Hospitalization and mortality through the six months follow-up period

Table 2 summarizes the characteristics of the dependent variables during six months of follow-up. Thirty-six patients (26.3%) were hospitalized for different causes. Of those, 6.6% were due to cardiac causes, and the rest were non-cardiac causes like chest infection (2.91%). Moreover, eight patients (5.83%) died; out of those, four patients (2.91%) were due to cardiac causes, and the rest were due to non-cardiac causes like infections (2.1%) and postoperative complications (0.7%).

**Table 2:** Hospitalization and mortality through 6 months follow up

**Variable**	**Frequency**	**Percentage (%)**
**Hospitalization**	36	26.3
**Cardiac**	9	6.6
**Non-cardiac**	27	19.7
Chest infection	4	2.91
Diabetic foot	2	1.46
Hypertensive emergency	2	1.46
Access problems	9	6.56
Others	11	8.03
**Mortality**	8	5.83
**Cardiac**	4	2.91
**Non-cardiac**	4	2.91
Infections	3	2.1
Post operation complications	1	0.7

Access problems (arteriovenous fistula repair, hematoma, infected catheter), others (post hysterectomy, squamous cell carcinoma, cellulitis, septic arthritis)

### Pathological synthesized ECG variables

Table 3 presents the descriptive analysis of the pathological synthesized ECG variables. Right ventricular Q wave was found in 3 patients (2.2%). Right ventricular ST elevation was found only in one patient (0.7%). Right ventricular negative T wave was found only in one patient (0.7%).

**Table 3:** Pathological Synthesized ECG Variables

**Variables**	**Yes (%)**	**No (%)**
**RV ST elevation**	1 (0.7)	136 (99.3)
**RV negative T wave**	1 (0.7)	136 (99.3)
**RV Q wave**	3 (2.2)	134 (97.8)

*ECG* Electrocardiogram, *RV* Right Ventricle

### The association between pathological synthesized ECG changes, mortality, cardiac and non-cardiac hospitalization

Fisher’s exact test was performed to examine the relation between pathological changes in synthesized ECG, mortality, cardiac and non-cardiac hospitalization among hemodialysis patients. While the correlation between pathological synthesized ECG changes and mortality was not significant in ESRD patients, (*p* > 0.999), the relation between pathological changes in synthesized ECG and hospitalization among hemodialysis patients was significant, (*p* = 0.047). Hospitalized patients due to non-cardiac causes were less likely to have pathological changes in the synthesized ECG, see Table 4.

**Table 4:** Pathological Changes in synthesized ECG and hemodialysis patients’ mortality and hospitalization (*N* = 137)

		**Pathological Changes in synthesized ECG**		**Fisher’s exact test**
	**Total n (%)** ***n* = 137**	**No n (%)** ***n* = 132**	**Yes n (%)** ***n* = 5**	**(*P* Value)**
**Mortality**				
None	129 (94.2)	124 (94)	5 (100)	> 0.999
Cardiac	4 (2.9)	4 (3)	0 (0.0)	
Non-Cardiac	4 (2.9)	4 (3)	0 (0.0)	
**Hospitalization**				
None	101 (73.7)	98 (74.2)	3 (60)	0.047
Cardiac	9 (6.6)	7 (5.3)	2 (40)	
Non-Cardiac	27 (19.7)	27 (20.5)	0 (0.0)	

*ECG* Electrocardiogram

## Discussion

This prospective cohort study aimed to assess the relationship between ECG variations in RV leads and predictable cardiovascular complications among ESRD patients on regular hemodialysis. The presence of pathological RV synthesized ECG changes was found to be associated with increased cardiac hospitalization in ESRD patients, and it was statistically significant (*p* = 0.047). Still, they are not associated with cardiac mortality (*p* = 0.9).

Data from previous studies indicate that the synthesized ECG can detect abnormalities concerning the right side of the heart with high sensitivity and specificity [33, 34]. Therefore, in this study, 18-leads synthesized ECG was used to detect RV lead pathological changes and the influence on hospitalization and mortality among ESRD patients.

Our analysis involved a total of 137 patients, and five patients were found to have any of the pathological RV ECG changes (RV Q wave, RV ST Elevation, and RV negative T wave). The presence of these changes was not significantly associated with increased mortality among ESRD patients. On the other hand, patients with pathological RV changes have a higher incidence of cardiac hospitalization (*p* = 0.007).

RVD and pulmonary hypertension are quite prevalent in patients on dialysis due to chronic pressure and volume overload, especially in the presence of brachial arteriovenous fistula [8, 35, 36]. In addition, these patients are usually prone to subclinical myocardial ischemia, which is ultimately caused by high volume removal and hemodynamic disturbances at hemodialysis sessions, which may also clarify the presence of pathological changes in RV leads. These subclinical ischemic events will result in further worsening of the RV function [37]. Moreover, LV function often worsens in combination with deterioration in RV function in dialysis patients as a consequence of ventricular interdependence [11, 38, 39].

As a result, congestive heart failure is widespread among ESRD patients, explaining the findings in our study. In addition, acute decompensated congestive heart failure was the most common cause of hospitalization in the nine patients who were hospitalized for cardiac causes during the 6-month follow-up period. Therefore, for ESRD patients, it is recommended for more regular HD sessions to avoid chronic overload and the use of a cooled dialysate to optimize hemodynamic parameters during the dialysis session [40–42]. These interventions will minimize subclinical ischemic events, preventing the occurrence of pulmonary hypertension and, as a result, prevent or delay the occurrence of RVD and LVD.

The diagnosis of right ventricle dysfunction and pulmonary hypertension is usually masked or missed in the ESRD population. These patients typically present with dyspnea, a common symptom in dialysis patients, usually attributed to other pathologies [43]. Synthesized ECG can be used as a simple, readily available, non-invasive screening tool for the early detection of pathological changes in RV leads. Further intervention, including more frequent HD sessions, could be carried out early based on these ECG findings to prevent RV function deterioration.

### Study strengths and limitations

Up to the authors’ knowledge, this study is the first of its type by correlating between synthesized ECG of right ventricular leads and cardiovascular complications. The outcomes of our study can be used as a framework for future studies to build on. In addition to that, the number of hemodialysis patients in this unit represents about 20% of all hemodialysis patients in the West Bank, Palestine. Therefore, the demographic, clinical, and biochemical characteristics of the hemodialysis patients included in this study are likely to reflect the hemodialysis population in Palestine. On the other hand, a 6-month follow-up period and relatively small sample size may not be enough to properly assess the relationship between pathological synthesized ECG changes, mortality, and cardiac and non-cardiac hospitalization. Drug history was not included in our study, and this should be taken into consideration in any further studies. Moreover, a correlation of these pathological ECG changes in RV leads with the echocardiographic evaluation of RV function is needed, which is not the case here.

## Conclusions

The presence of pathological RV ECG abnormalities could predict cardiac hospitalization in ESRD patients. Synthesized ECG is a good available tool that can easily be performed in ESRD patients to detect pathological RV changes. RV pathological abnormalities, on the other hand, are a rare occurrence. Further study with a high number of patients and a longer follow-up time is needed to determine whether Synthesized ECG can be employed as a screening tool for pathological RV abnormalities in dialysis patients.

## Data Availability

All data supporting the study are presented in the manuscript or available upon request from the manuscript’s corresponding author.

## Abbreviations

ESRD: End-Stage Renal Disease
PMP: Per Million Population
CVD: Cardiovascular Disease
LVD: Left Ventricular Dysfunction
RVD: Right Ventricular Dysfunction
RVF: Right Ventricular Function
HD: Hemodialysis
CMR: Cardiac Magnetic Resonance
ECG: Electrocardiogram
QTc: Corrected QT
RV: Right Ventricle
MI: Myocardial Infarction
IRB: Institutional Review Board
SD: Standard Deviation
SPSS: Statistical Package of Social Sciences
UF: Ultrafiltration
CI: Confidence Interval
OR: Odds Ratio
